# Role of Neoadjuvant Immunotherapy in Genitourinary Malignancies

**DOI:** 10.3390/cancers16244127

**Published:** 2024-12-10

**Authors:** Adam Khorasanchi, Karan Jatwani, Lingbin Meng, Katharine A. Collier, Debasish Sundi, Shawn Dason, Eric A. Singer, Dharmesh Gopalakrishnan, Amir Mortazavi, Gurkamal Chatta, Yuanquan Yang

**Affiliations:** 1 Division of Hospital Medicine, The Ohio State University Comprehensive Cancer Center, Columbus, OH 43210, USA; adam.khorasanchi@osumc.edu; 2 Department of Medicine, Roswell Park Comprehensive Cancer Center, Buffalo, NY 14263, USA; jatwani.karan@gmail.com (K.J.); dharmesh.gopalakrishnan@roswellpark.org (D.G.); gurkamal.chatta@roswellpark.org (G.C.); 3 Division of Medical Oncology, The Ohio State University Comprehensive Cancer Center, Columbus, OH 43210, USA; lingbin.meng@osumc.edu (L.M.); katharine.collier@osumc.edu (K.A.C.); amir.mortazavi@osumc.edu (A.M.); 4 Division of Urologic Oncology, The Ohio State University Comprehensive Cancer Center, Columbus, OH 43210, USA; d.sundi@osumc.edu (D.S.); shawn.dason@osumc.edu (S.D.); eric.singer@osumc.edu (E.A.S.); 5 Pelotonia Institute for Immuno-Oncology, The Ohio State University Comprehensive Cancer Center, Columbus, OH 43210, USA

**Keywords:** neoadjuvant, immunotherapy, immune checkpoint inhibitors, urothelial carcinoma, renal cell carcinoma, prostate cancer, testicular germ cell tumors, penile squamous cell carcinoma

## Abstract

Genitourinary (GU) malignancies are common and associated with significant morbidity and mortality. In patients with localized GU cancers, surgical resection or definitive radiation remain the mainstays of treatment. Despite definitive treatment, many patients with high-risk localized disease experience recurrence. There is growing interest in using neoadjuvant immunotherapy to improve outcomes. This narrative review summarizes the current evidence for neoadjuvant immunotherapy in patients with localized high-risk GU cancers including renal cell carcinoma, urothelial carcinoma, prostate cancer, penile squamous cell carcinoma, and testicular germ cell tumors. We also discuss ongoing clinical trials and candidate biomarkers to optimize patient selection and improve treatment outcomes.

## Simple Summary

Immune checkpoint inhibitors (ICIs), which can improve the immune system’s ability to recognize and eliminate cancer cells, have significantly improved outcomes for patients with advanced kidney and bladder cancer. This success has prompted further research into the use of ICIs prior to definitive treatment in patients with localized genitourinary tumors at high risk for reoccurrence. Preliminary results from kidney and bladder cancer studies appear promising; however, further research is needed to evaluate a larger number of patients to demonstrate a survival benefit (compared to standard treatments) prior to the adoption of these treatment strategies. Conversely, the use of ICIs prior to definitive treatment thus far has demonstrated limited effectiveness in prostate cancer, likely due to dampening of the immune system response by the tumor and its surrounding cells. Overcoming these immune-suppressive mechanisms will require the development of biological tools that can better predict treatment response. This remains an area of active investigation.

## 1. Introduction

Genitourinary (GU) malignancies are a heterogeneous group of biologically distinct tumors associated with significant morbidity and mortality [1]. Prostate adenocarcinoma (PC); urothelial carcinoma (UC) of the bladder, ureter, and renal pelvis; and renal cell carcinoma (RCC) are the most common types [2]. In patients with localized GU cancers, surgical resection or radiation therapy (RT) represent the cornerstones of treatment [3,4,5]. Unfortunately, a significant proportion of patients with high-risk disease experience recurrence, which is often associated with a poor prognosis [6,7,8]. Currently, there is growing interest in the use of neoadjuvant therapies to minimize the risk of disease recurrence and improve survival outcomes. In addition to improving oncologic outcomes, neoadjuvant systemic therapy may also provide additional advantages from a surgical perspective, including improved resectability of locally advanced tumors.

While the use of neoadjuvant therapies in RCC and PC remains investigational, cisplatin-based combination neoadjuvant chemotherapy (NAC) is the current standard of care (SoC) in muscle-invasive bladder cancer (MIBC). However, NAC confers an absolute 5-year overall survival (OS) benefit of only 5–8% [9,10], and its use remains limited as about half of MIBC patients at the time of their diagnosis are cisplatin-ineligible (cis-ineligible) due to comorbidities and performance status [11,12]. Given the recent success of immune checkpoint inhibitors (ICIs) in the treatment of metastatic RCC (mRCC) and metastatic UC (mUC), there is significant interest in the use of ICIs in the neoadjuvant setting [13].

ICIs have already been incorporated in the perioperative management of GU cancers. Specifically, adjuvant ICIs are the SoC treatment for high-risk localized RCC and may benefit select patients with UC [14,15]. Furthermore, there is evidence to suggest that a perioperative approach may be effective due to tumor-draining lymph nodes acting as a reservoir for neoantigens, enabling greater T-cell priming and an augmented antitumor immune response [14,16,17,18,19,20].

In this review, we summarize the current evidence for neoadjuvant immunotherapy in patients with localized high-risk GU cancers (including node positive, T4M0 disease). We also briefly discuss rare testicular and penile cancers, for which there are more limited data on neoadjuvant immunotherapy. Finally, we discuss ongoing neoadjuvant immunotherapy clinical trials and emerging biomarkers designed to optimize patient selection and improve outcomes for these patients.

## 2. Methodology

**Figure 1:**
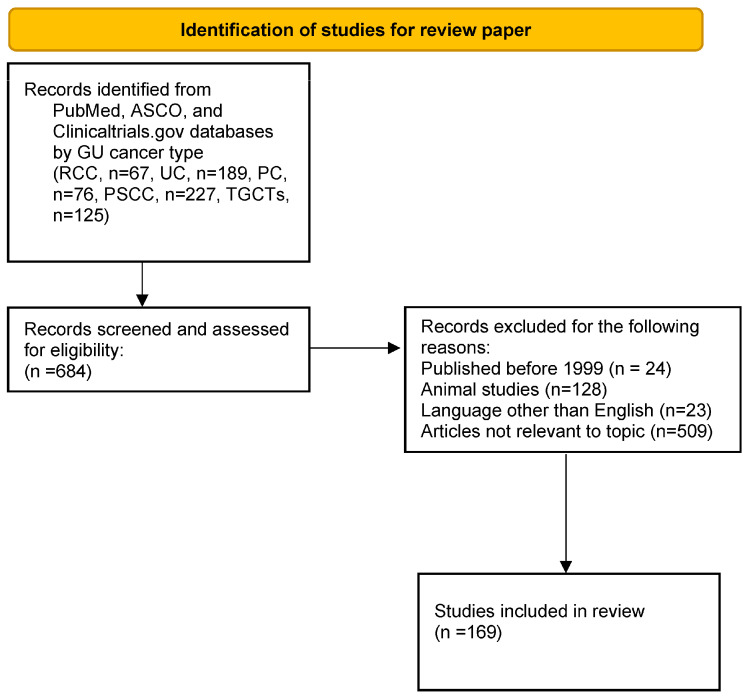
PRISMA flow diagram of literature search and study selection for review.

A literature search identified relevant articles in the PubMed, American Society of Clinical Oncology (ASCO), and clinicaltrials.gov databases. The search terms “neoadjuvant”, “immunotherapy”, “immune checkpoint inhibitors”, “renal cell carcinoma”, “urothelial carcinoma”, “prostate cancer”, “penile cancer”, and “testicular germ cell tumors” were used. The search revealed papers published between the years 1971 and 2024 according to the following GU cancer types: RCC (*n* = 67), UC (*n* = 189), PC (*n* = 76), penile squamous cell carcinoma (PSCC) (*n* = 227), and testicular germ cell tumors (TGCTs) (*n* = 125). These papers were screened for eligibility. Only full-text articles written in English and published between the years 2001 and 2024 were included. Articles that did not involve human subjects were excluded. Articles not relevant to this topic (*n* = 509) were excluded. A total of 169 articles were included in this review (Figure 1).

## 3. Neoadjuvant Immunotherapy in Patients with High-Risk RCC

### 3.1. Rationale for Investigating Neoadjuvant Immunotherapy in Patients with High-Risk RCC

Surgical resection, via radical or partial nephrectomy (PN), represents the SoC for patients with localized RCC [3]. Despite curative intent, a significant proportion of patients with high-risk RCC (40–80%) experience disease recurrence [21]. While there is no universally accepted definition of high-risk disease, clinicopathologic features that may influence prognosis include tumor stage, grade, sarcomatoid differentiation, the presence of necrosis, and performance status [22]. Identifying patients with localized high-risk RCC is more challenging in the neoadjuvant setting as several of these prognostic factors rely on histopathologic examination of the excised tumor, which is unknown before surgery [23,24].

**Figure 2:**
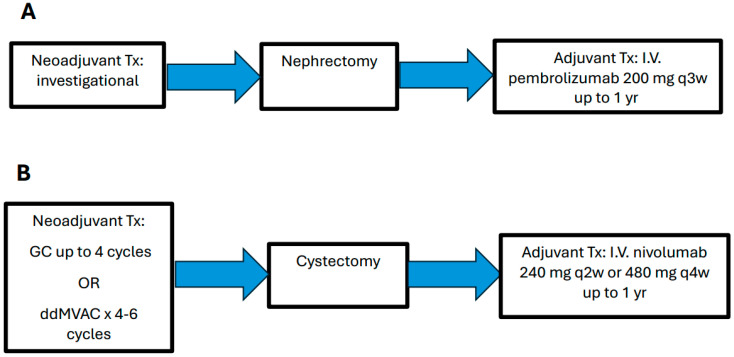
Current perioperative management of localized high-risk GU cancers. RCC (**A**), UC (**B**).

ICI-based combination therapies currently represent the mainstay of treatment in mRCC [25]. Given the success of ICIs in the treatment of advanced RCC, this has prompted further investigation into the use of perioperative immunotherapy to reduce the risk of recurrence and improve survival outcomes [8,20]. For instance, adjuvant pembrolizumab is currently approved for use in patients with localized high-risk clear cell RCC (ccRCC) based on results from the KEYNOTE-564 trial, which demonstrated improved disease-free survival (DFS) and OS (Figure 2) [18,26].

From a surgical standpoint, a neoadjuvant immunotherapy approach in RCC may offer several advantages. These include (1) tumor downstaging, leading to improved surgical resectability and preservation of adjacent organs; (2) facilitating PN, therefore preventing the need for renal replacement therapy; and (3) reducing inferior vena cava (IVC) tumor thrombus burden, thus minimizing surgical morbidity risk and the need for vascular reconstruction [27,28]. From an oncologic standpoint, a neoadjuvant immunotherapy approach may enable a more robust antitumor immune response on a tumor in situ due to increased T-cell expansion and activity [13,29]. The neoadjuvant setting also uniquely allows for the collection of valuable tissue samples before and after treatment, aiding in biomarker discovery and validation, which is critical for identifying patients most likely to respond to ICIs. Finally, early assessment of treatment efficacy through pathological assessment, imaging, and biomarker studies can guide more informed treatment decisions and reduce unnecessary exposure to ineffective therapies [29]. However, there are also potential risks associated with neoadjuvant immunotherapy. These risks may include (1) the overtreatment of patients whose disease can be cured with surgery alone; (2) disease progression (PD) during neoadjuvant treatment, which may result in a lost opportunity for cure via surgical resection; (3) the harboring of immune-suppressive cells in the primary tumor that may mitigate immunotherapy response; (4) the development of complications such as tumor-related bleeding or venous tumor thrombus; (5) the onset of immune-related adverse events (irAEs), which can potentially delay or jeopardize curative surgery; (6) the treatment of irAEs with immunosuppressants, which can cause further delays in surgical treatment; and (7) the development of inflammatory tissue changes and fibrosis, which can increase the risk of surgical complications [30,31,32,33].

### 3.2. Clinical Trials of Neoadjuvant Immunotherapy in High-Risk RCC (Table 1)

#### 3.2.1. Nivolumab Monotherapy

To date, there has been one phase 1, one phase 2, and one phase 3 trial of neoadjuvant nivolumab for localized high-risk RCC. The first was a phase 1 primary safety/tolerability study, which evaluated 17 patients with nonmetastatic high-risk (clinical stage T2a–T4 and/or N1) ccRCC who were treated with nivolumab every 2 weeks for three doses before surgery [34]. All patients underwent surgery without delay. Ten patients (58.8%) developed grade 1–2 treatment-related AEs (trAEs); no grade 4–5 trAEs were observed. All evaluable patients treated with nivolumab were noted to have stable disease (SD) per Response Evaluation Criteria in Solid Tumors (RECIST) during treatment, while one patient demonstrated a pathologic response suggestive of possible antitumor activity. Median follow-up was 2 years with metastasis-free survival (MFS) and OS rates of 85% at 3 years. Next, a phase 2 trial evaluated 18 patients with locally advanced ccRCC who were treated with up to four doses (8 weeks) of neoadjuvant nivolumab. Again, the best response for all patients was SD per RECIST. All patients completed surgery without delay; however, two patients discontinued treatment due to irAEs, and four patients developed surgical complications. Median follow-up was 23 months, and the median recurrence-free survival (RFS) at 1 year was 82%. At least a 5% pathological tumor regression was noted in 10 of 14 evaluable cases when comparing the pre-nivolumab tumor samples and post-nivolumab nephrectomy specimens [35]. Finally, the phase 3 PROSPER trial evaluated 819 patients with high-risk RCC (≥cT2; N+ or oligometastatic disease that could be rendered no evidence of disease within 12 weeks of surgery) who were randomized to perioperative nivolumab followed by nephrectomy or nephrectomy alone, regardless of tumor histology [36]. Patients on the experimental arm were treated with one dose of neoadjuvant nivolumab followed by nine doses of adjuvant nivolumab. This trial was terminated early due to futility as the primary endpoint, median RFS, was not reached, with similar numerical results between the study arms. OS data were immature at the time of analysis, and numerical results were also similar between the two arms. Twenty percent of patients treated with perioperative nivolumab had at least one grade 3–4 AE versus 6% of patients in the control arm [37]. The negative results of the PROSPER trial may be due to its heterogenous patient population, as pathologic downstaging of cT2 patients could have led to the inclusion of a significant proportion of patients with a lower risk of disease recurrence. Additionally, non-ccRCC patients were included; the data on ICI efficacy are more limited in this group. The trial design may also have led to disproportionate treatment and follow-up between the study arms, as well as inadequate nivolumab exposure—only 53% in the experimental group received the full course of therapy. Finally, patients only received one dose of neoadjuvant nivolumab, which may have been insufficient to generate an effective antitumor immune response [38].

**Table 1:** Summary of completed prospective neoadjuvant immunotherapy trials in high-risk RCC.

NCT Number	Pt Selection	Phase	Est. pts	Trial Title	Sponsor	Ref.
NCT02575222	cT2a-T4Nany; or cTanyN1	I	17	Neoadjuvant Nivo in pts with High-risk Nonmetastatic RCC	Hopkins	[34]
NCT02595918	ccRCC	II	19	Nivo in Treating pts With High-Risk Kidney Cancer Before Surgery	NCI	[35]
NCT03055013	≥cT2NanyM0; cTanyN + M0; or oligomets that could be rendered NED	III	819	Nivo in Treating pts With Localized Kidney Cancer Undergoing Nephrectomy	NCI	[37]
NCT02762006	cT2b-4 and/or N1	Ib	29	Periop durva +/− tremelimumab in locally advanced RCC	Case	[39]
NCT03680521	cT2b-3bN0	II	25	Neoadjuvant Sitravatinib + Nivo in pts With ccRCC	Mirati Therapeutics	[40]
NCT05172440	cT2a-4 and/or N1	II	20	A Study on the Safety and Effectiveness of Tislelizumab + Axitinib for Neoadjuvant Tx of ccRCC	The Affiliated Nanjing Drum Tower Hospital of Nanjing University Medical School	[41]
NCT04118855	cT2-T3N0-1	II	20	Toripalimab + Axitinib as Neoadjuvant Tx in pts With Non-metastatic Locally Advanced Nonmetastatic ccRCC	RenJi Hospital	[42]

Abbreviations: NCT, national clinical trial; pt, patient; est, estimated; ref, reference; nivo, nivolumab; RCC, renal cell carcinoma; oligomets, oligometastatic disease; NED, no evidence of disease; periop, perioperative; durva, durvalumab; ccRCC, clear cell RCC; Tx, treatment.

In summary, the clinical trials described showed that neoadjuvant nivolumab can be administered safely, and there may be pathologic signs of an immune response. However, the tumor downstaging effects were limited. A longer treatment duration may be associated with a higher response rate. Finally, PROSPER, the only phase 3 neoadjuvant single-agent ICI trial to date, failed to demonstrate improvement in patient outcomes (RFS or OS).

#### 3.2.2. ICI/ICI Combination Therapies

Based on the results of neoadjuvant single-agent ICI therapy, ICI combination therapies were investigated in RCC. A phase 1b safety/feasibility trial evaluated neoadjuvant/adjuvant use of the anti-PD-ligand 1 (anti-PD-L1) durvalumab +/− the anti-cytotoxic lymphocyte antigen-4 (anti-CTLA-4) tremelimumab. Preliminary results were presented at the 2020 GU ASCO meeting. Twenty-nine patients with localized high-risk ccRCC (cT2b–4 and/or N1) were divided into four perioperative treatment arms. Patients were treated with a single dose of neoadjuvant treatment followed by either one dose or up to 1 year of adjuvant therapy, dependent on the assigned treatment arm. While there were no reported surgical delays or associated complications, patients experienced a higher-than-expected rate of irAEs, resulting in the early termination of the study. There was no significant tumor downstaging with neoadjuvant immunotherapy; however, only one cycle was given before nephrectomy [39]. In summary, the use of ICI/ICI combination therapy in this single trial was limited by its toxicity profile.

#### 3.2.3. ICI/TKI Combination Therapies

There have been three phase 2 trials of neoadjuvant ICI + tyrosine kinase inhibitor (TKI) combination regimens. The first was a phase 2 study that evaluated 17 patients with locally advanced ccRCC (cT2-3bN0) who were treated for up to 8 weeks with neoadjuvant nivolumab + sitravatinib, a novel, multitarget TKI [40]. The primary endpoint, objective response rate (ORR), was not met (11.8% [95% confidence interval (CI), 1.5–36.4%; *p* = 0.208]), as it was below the prespecified target of 30%. Two patients discontinued nivolumab due to grade 2 pneumonitis and thyroiditis. There were no reported grade 4–5 trAEs, and the 2-year DFS was 88% (95% CI, 61.0–97.0%). Next, a single-arm phase 2 trial evaluated the use of neoadjuvant tislelizumab (anti-PD-1) + axitinib, a small-molecule TKI, in 20 patients with high-risk localized ccRCC (cT2a-4 and/or N1) [41]. Eighty-four percent (11/13) of the patients completed 12 weeks of neoadjuvant therapy, and 69% (9/13) underwent surgery without delay. One patient experienced PD during treatment, requiring further systemic therapies. Of the nine evaluable patients, the investigator-assessed confirmed ORR was 55.5%. There were no intraoperative or post-surgical complications. Additionally, a single-arm phase 2 trial evaluated 20 patients with locally advanced ccRCC treated with axitinib + toripalimab (anti-PD-1) for up to 12 weeks. Fifteen of the nineteen patients (79%) underwent surgery without delay, and the primary endpoint, ORR, was 45%. Tumor shrinkage was observed in 95% of the patients before surgical resection, and four patients (21%) achieved a pathological complete response (pCR). Grade ≥ 3 AEs occurred in 25% of the patients, of which one patient discontinued treatment and received intravenous (IV) hydrocortisone due to grade 3 transaminitis [42]. Finally, a single-arm phase 2 trial (NCT03341845) is currently evaluating the use of 12 weeks of neoadjuvant avelumab (anti-PD-L1) + axitinib in patients with high-risk ccRCC (cT1b–4N0 or cTanyN1). Initial results reported at the 2022 GU ASCO meeting were positive: 12/40 (30%) patients achieved partial response (PR) per RECIST. Among the post-treatment samples of patients who experienced disease recurrence, there was a significantly reduced CD8+ density. There were no treatment-related surgery delays or PD observed [43]. Eight of the forty patients (20%) experienced postoperative AEs. Median follow-up was 2 years, recurrence occurred in 13/40 (32%) patients, and three deaths were reported. Eleven of the twelve patients (92%) who achieved PR had no evidence of disease at the time of initial trial reporting. This study is ongoing with data on the secondary endpoints, DFS and OS, forthcoming.

In summary, neoadjuvant ICI/TKI combination therapies showed promising antitumor activity in patients with high-risk RCC; however, several questions remain, including optimal patient selection, treatment duration, and the impact of neoadjuvant ICI/TKI combination therapies on long-term and overall survival outcomes.

## 4. Neoadjuvant Immunotherapy in Patients with Muscle-Invasive UC

### 4.1. Rationale for Investigating Neoadjuvant Immunotherapy in Patients with Muscle-Invasive UC

UC accounts for a majority (90%) of bladder cancer cases. Patients with localized MIBC are at high risk for recurrence and may benefit from perioperative systemic therapy to treat microscopic disease [44]. Currently, cisplatin-based NAC represents the SoC for eligible MIBC patients before radical cystectomy (RC) [4,9]. The two most commonly used NAC regimens are gemcitabine and cisplatin (GC) and dose-dense methotrexate, vinblastine, adriamycin, and cisplatin (ddMVAC) (Figure 2) [45]. pCR is often used as a surrogate endpoint in neoadjuvant trials [46]. ddMVAC has consistently shown higher ORR and pCR rates than GC in retrospective studies and cross-study comparisons [47,48]. Furthermore, the only direct comparison of these two regimens was in the VESPER trial, in which pCR rates were 42% with ddMVAC and 36% with GC [49,50]. Additionally, a phase 2 study evaluating MIBC patients (cT2-4a, N0-1) demonstrated a 38% pCR rate following only three cycles (6 weeks) of ddMVAC, which showed similar efficacy to the previously utilized 12-week regimen [51]. Irrespective of which NAC regimen is utilized, patients achieving ypT0N0 or ypT1N0 status have superior survival outcomes: 85% at 5 years [52,53,54].

However, several challenges exist for the use of NAC in patients with MIBC: nearly half of the patients are cis-ineligible, there is an increased risk of toxicity, responses to treatment are variable, and it can potentially delay RC [11,55]. This has prompted the investigation of ICIs in the perioperative setting, given their favorable tolerability profile and the possibility of durable responses.

Currently, ICI-based therapies represent the SoC for mUC and have already been successfully incorporated as the SoC adjuvant treatment for localized high-risk MIBC (Figure 2) [4,56]. The CheckMate 274 trial demonstrated improved DFS in patients treated with adjuvant nivolumab compared to surgery alone. Interim OS data favored nivolumab versus the placebo in the intention-to-treat analysis (hazard ratio [HR] 0.76, 95% CI 0.61–0.96) and tumor PD-L1 >1% populations (HR 0.56, 95% CI 0.36–0.86) [14,57,58]. Additionally, in the phase 3 AMBASSADOR trial (Alliance A031501), adjuvant pembrolizumab demonstrated improved DFS compared to observation alone in patients with high-risk MIBC (≥pT3, pN+, + margins for cis-ineligible patients, and ≥ypT2, yN+, + margins for patients treated with NAC) [15]. Finally, adjuvant atezolizumab in the IMvigor010 trial failed to demonstrate improved DFS in patients with high-risk MIBC (pT3-4a, or pN+ for cis-ineligible patients, and ypT2-4a, or ypN+ for patients treated with NAC) [59].

As previously stated, a neoadjuvant treatment approach may be efficacious in localized disease due to a higher tumor antigen load resulting in a more effective antitumor immune response via enhanced T-cell priming and activation [60]. For cis-eligible patients with high-risk MIBC, efforts are currently underway to investigate the addition of neoadjuvant ICI to chemotherapy to improve the pCR or reduce toxicity by replacing NAC with ICI. For cis-ineligible patients, ICI may provide a neoadjuvant option, which does not currently exist.

### 4.2. Clinical Trials of Neoadjuvant Immunotherapy in Muscle-Invasive UC (Table 2)

#### 4.2.1. ICI Monotherapy

There have been one phase 1b and three phase 2 trials evaluating neoadjuvant ICI monotherapy in MIBC. First, the phase 2 PURE-01 trial evaluated the use of three cycles of neoadjuvant pembrolizumab in 50 MIBC patients (cT ≤ 3bN0), regardless of cisplatin eligibility, though 90% of the patients in this study were cis-eligible [61]. Preliminary results demonstrated that 42% of the patients achieved the primary endpoint of the pCR (ypT0), and 54% experienced tumor downstaging (ypT < 2). Tumors with a PD-L1 combined positive score >10 or high tumor mutational burden (TMB) correlated with an increase in the observed pCR [62]. A follow-up of the PURE-01 trial included 155 patients in the intention-to-treat population, with a median follow-up time of 39 months. The key findings included a 36-month event-free survival (EFS) and OS of 74.4% [95% CI, 67.8–81.7%] and 83.8% (95% CI, 77.8–90.2%), respectively. The pCR in this cohort was 37% [63]. Thirty-four percent of the patients experienced high-grade surgical complications (Clavien-Dindo ≥ 3a) with no perioperative mortality at 90 days. Next, the phase 2 ABACUS trial investigated two cycles of the PD-L1 inhibitor atezolizumab for the treatment of 88 cis-ineligible MIBC patients (cT2-4aN0) prior to RC [64]. The study met its primary endpoint, as the pCR was 31% (95% CI, 21–41%). Grade 3–4 trAEs occurred in 11% of the patients, and there were no surgical delays [65]. Grade 1–2 surgical complications were observed in 45% of the patients; 17% experienced grade 3–4 AEs. Two-year DFS and OS were 68% (95% CI 58–76) and 77% (95% CI 68–85), respectively. The two-year DFS in patients achieving a pCR was 85% (95% CI 65–94) [66]. Next, the use of four cycles of neoadjuvant avelumab +/− gemcitabine in 56 cis-ineligible patients with MIBC (cT2-4aN0-2) was investigated in the phase 2 AURA trial [67]. This study demonstrated a pCR of 36% in those treated with avelumab monotherapy (*n* = 28). Finally, the phase 1b trial PrE0807 evaluated the feasibility of two doses of neoadjuvant nivolumab +/− lirilumab (anti-killer immunoglobulin receptor) in 43 cis-ineligible patients with MIBC (cT2-4aN0-1) [68]. All 13 patients in the nivolumab monotherapy cohort completed treatment and 12/13 underwent RC. The pCR rate was only 8% and the proportion of patients who achieved pathologic downstaging was 17%. There were no grade 3 trAEs in those who treated with nivolumab alone.

**Table 2:** Summary of completed prospective neoadjuvant immunotherapy trials in muscle-invasive UC.

NCT Number	Pt Selection	Phase	Est. pts	Trial Title	Sponsor	Ref.
NCT02736266	cT ≤ 3bN0	II	174	Neoadjuvant Pembro for MIBC (PURE-01)	Fondazione IRCCS Istituto Nazionale dei Tumori, Milano	[61]
NCT03674424	cT2-4aN0-2	II	137	Avelumab as Neoadjuvant Tx in Subjects With MIBC (AURA Trial)	Jules Bordet Institute	[67]
NCT02989584	cT2-T4aN0	II	54	Atezo + Cisplatin + Gemcitabine Before Surgery to Remove the BC	MSKCC	[69]
NCT03387761	cT3-4aN0; or cT1-4aN1-3	II	54	Neo-Adjuvant Bladder UC COmbination-immunotherapy (NABUCCO)	The Netherlands Cancer Institute	[70]
NCT02812420	cT2-3aN0	I	54	Durva + Tremelimumab in Treating pts With High-Risk MIBC That Cannot Be Tx With Cisplatin-Based Tx Before Surgery	MDACC	[71]
NCT03472274	cT2-T4aN ≤ 1	II	101	Durva + TREmelimumab in NEOadjuvant BC Pts (DUTRENEO)	Fundacion CRIS de Investigación para Vencer el Cáncer	[72]
NCT03558087	cT2-T4aN0	II	76	GC + nivo with selective bladder sparing in pts with MIBC	Matthew Galsky	[73]
NCT03294304	cT2-T4a, N ≤ 1	II	41	BC Signal Seeking Trial of nivo, GC, in MIBC undergoing cystectomy	University of Minnesota	[74]
NCT02690558	cT2-4a N0/X	II	39	GC + pembro as neoadjuvant tx prior to RC in pts with MIBC	UNC	[75]
NCT02365766	cT2-4aN0	Ib/II	83	Neoadjuvant Pembro in Combination With Gemcitabine Tx in Cis-eligible/Ineligible UC pts	Jason R. Brown	[76]
NCT03406650	cT2-T4a N0-1	II	61	Periop Chemoimmunoth-erapy With Durva for MIBC	Swiss Group for Clinical Cancer Research	[77]
NCT03549715	cT2-4N0-1	I/II	121	Durva +/− tremelimumab in combination with ddMVAC as neoadjuvant tx in pts with MIBC	Association Pour La Recherche des Thérapeutiques Innovantes en Cancérologie	[78]
NCT03532451	cT2-4aN0-1	Ib	43	Neoadjuvant nivo +/− lirilumab in cis-ineligible pts with MIBC	PrECOG	[68]
NCT03732677	cT2-T4aN0/1	III	1063	Durva+ GC (Neoadjuvant Tx) and Durva (Adjuvant Tx) in Pts With MIBC	AstraZeneca	[79]

Abbreviations: NCT, national clinical trial; pt, patient; est, estimated; ref, reference; pembro, pembrolizumab; MIBC, muscle-invasive bladder cancer; atezo, atezolizumab; durva, durvalumab; tx, treatment; GC, gemcitabine + cisplatin; nivo, nivolumab; RC, radical cystectomy; cis, cisplatin; UC, urothelial carcinoma; periop, perioperative; ddMVAC, dose-dense methotrexate, vinblastine, doxorubicin, cisplatin.

In summary, the PURE-01 trial was one of the largest trials evaluating neoadjuvant single-agent ICI in patients with MIBC. Neoadjuvant pembrolizumab demonstrated an acceptable safety profile, as AE rates were comparable to those reported following NAC [80]. Another key finding of this trial was the association between PD-L1 expression and improved pCR rates, suggestive of the potential role of PD-L1 expression as a biomarker for antitumor immune response. However, this finding was not seen in the ABACUS trial, potentially due to differences in the assays used, the types of ICI utilized, and the patient cohorts [81]. While these early-phase studies demonstrated the potential role of single-agent neoadjuvant ICI in the management of patients with MIBC, larger phase 3 studies are needed to prove a survival benefit.

#### 4.2.2. Chemoimmunotherapy

Following the results of single-agent neoadjuvant ICI, ICI combination therapies were investigated in patients with MIBC. There have been several phase 2 trials and one phase 3 trial of neoadjuvant chemoimmunotherapy combination regimens. First, as previously mentioned, the use of neoadjuvant avelumab +/− gemcitabine was investigated in the phase 2 AURA trial [67]. Those treated with avelumab + gemcitabine (*n* = 28) had a pCR of 18%, which suggests that the addition of gemcitabine may reduce avelumab efficacy. Of note, two patients in the chemoimmunotherapy arm experienced grade 3 irAEs (hepatitis and pneumonitis), one of which led to ICI discontinuation. Second, a phase 2 trial evaluated six doses of neoadjuvant atezolizumab + GC in 39 patients with MIBC (cT2-4aN0) [69]. The study met its primary endpoint, as 69% (27/39) achieved tumor downstaging, including 41% (16/39) achieving pCR. Grade 3 irAEs occurred in five (11%) patients, with two (5%) patients requiring systemic steroids. Third, the phase 1b/2 HCRN GU14-188 trial investigated 82 patients with MIBC (cT2-4aN0) treated with five doses of neoadjuvant pembrolizumab and gemcitabine +/− cisplatin [76]. Cis-eligible (*n* = 42) and cis-ineligible (*n* = 40) patient cohorts were included. Both cohorts demonstrated significant tumor downstaging (41%). The rate of ≥grade 3 irAEs was low, with the following toxicities reported: transaminitis (3.7%), rash (2.5%), pneumonitis (2.5%), and colitis (2.5%). Next, the phase 2 BLASST-1 trial demonstrated significant pathologic downstaging in 41 patients with MIBC (cT2-4a, N ≤ 1) treated with at least one dose (up to 4 cycles) of neoadjuvant nivolumab and GC [74]. Notably, <1% of the patients developed irAEs; one patient was treated for Guillain-Barre syndrome with IV immunoglobulin (IVIg), and none required steroids. Additionally, the phase 2 HCRN GU16-257 trial evaluated five doses of neoadjuvant nivolumab plus GC as organ-sparing treatment for 76 cis-eligible patients with MIBC (cT2-4aN0) [73]. Thirty-three patients (43%) achieved a clinical CR rate (cCR) following chemoimmunotherapy, of which 32 patients chose to forgo immediate RC. This was associated with significantly improved MFS and OS. Subsequently, the phase 2 SAKK 06/17 trial evaluated the addition of perioperative durvalumab (four cycles neoadjuvant, four cycles adjuvant) in combination with neoadjuvant GC in 57 cis-eligible patients with MIBC (cT2-T4a, N0-1) [77]. Thirty-three percent (17/52) of the patients achieved pCR and 60% (31/52) had a pathologic response (<ypT2N0). This study also demonstrated an improved EFS of 76% (95% CI = 62–85%), and OS was 85% (95% CI = 72–92%) at 2 years. Seven patients (13%, *n* = 57) developed any-grade irAEs during neoadjuvant treatment, five of which were ≥grade 3. Next, a phase 2 trial evaluated the use of four cycles of neoadjuvant ddMVAC in combination with two cycles of durvalumab +/− tremelimumab in 119 patients with MIBC (cT2-4N0-1) [78]. The pCR was similar in both arms: 49% with ddMVAC + durvalumab (*n* = 55) and 47% with ddMVAC + durvalumab + tremelimumab (*n* = 58). Pathologic downstaging occurred in 66% of the patients overall. All of the patients developed at least one trAE; ≥grade 3 trAEs were present in 49 patients (41%). Additionally, a phase 2 study evaluated neoadjuvant gemcitabine and split-dose cisplatin + pembrolizumab in 39 patients with MIBC (cT2-4aN0) [75]. The study met its primary endpoint, as 22/39 (56%) of the patients experienced pathologic downstaging. One patient developed ICI-associated diabetic ketoacidosis, which did not require steroid use. Finally, the phase 3 NIAGARA (NCT03732677) trial evaluated the use of perioperative durvalumab + GC in cis-eligible patients with MIBC (cT2-TaN0/1) [79]. Patients (*N* = 1063) were randomized (1:1) to either four cycles of neoadjuvant durvalumab + GC and eight cycles of adjuvant durvalumab following RC (*n* = 533) or neoadjuvant GC followed by RC alone (*n* = 530). There were no treatment-related delays to surgery, and perioperative durvalumab + NAC resulted in significant improvements in EFS and OS compared to the SoC treatment. In the perioperative durvalumab treatment arm, 111 patients (21%) developed irAEs. The incidence of irAEs reported in the treatment arm were as follows: hypothyroidism (10.4%), hyperthyroidism (2.5%), dermatitis (2.3%), nephritis (1.7%), colitis (1.5%), and pneumonitis (1.3%). None of these irAEs led to death.

In summary, several of these phase 2 studies demonstrated that neoadjuvant chemoimmunotherapy in patients with MIBC was well tolerated and associated with significant tumor downstaging. The HCRN GU16-257 study was unique, as it evaluated the role of neoadjuvant chemoimmunotherapy in definitive bladder-sparing treatment for patients with MIBC. Additionally, unlike other studies, cCR was used as a primary endpoint and was found to be associated with improved survival outcomes. Finally, based on the positive results of the phase 3 NIAGARA trial, perioperative chemoimmunotherapy offers the potential to be incorporated as the new SoC for cis-eligible patients with MIBC.

#### 4.2.3. ICI/ICI Combination Therapies

There have been four phase 2 trials evaluating the use of neoadjuvant ICI/ICI combination therapies in MIBC. The feasibility of two doses of neoadjuvant nivolumab 1mg/Kg + ipilimumab 3mg/Kg was investigated in the single-arm NABUCCO trial [70]. Twenty-four patients with stage III UC (cT3-4aN0 or cT1-4aN1-3) who were either cis-ineligible or declined chemotherapy were enrolled; ten of these patients had baseline lymph node metastases (cN1-3). The primary endpoint was surgical resection within 12 weeks of treatment initiation. All of the patients underwent surgery, with 96% doing so within 12 weeks. Eleven patients (46%) achieved a pCR, and downstaging was demonstrated in 58% of the patients. Notably, this trial reported a higher proportion of grade 3–4 irAEs (55%) compared to other neoadjuvant ICI trials, likely due to the use of combination ICI agents. Next, the phase 2 University of Texas MD Anderson Cancer Center (MDACC) trial (NCT02812420) evaluated two doses of neoadjuvant durvalumab + tremelimumab in 28 cis-ineligible patients with high-risk UC [71]. High-risk UC was defined by features of bulky T3/T4 tumors, variant histology, lymphovascular invasion, hydronephrosis, and/or high-grade upper tract disease. The pCR was 37.5%, and there was downstaging to ≤pT1 in 58% of the patients who completed surgery. Twenty-one percent of the patients experienced grade ≥3 irAEs. Additionally, the phase 2 DUTRENEO trial evaluated three cycles of neoadjuvant durvalumab + tremelimumab vs. SoC cisplatin-based chemotherapy. Sixty-one high-risk (cT2-T4aN ≤ 1) operable cis-eligible patients with UC were further subdivided into “hot” and “cold” tumor groups based on an 18-gene interferon (IFN)-gamma signaling-based tumor inflammation signature (TIS) [72]. Patients in the “cold” tumor group received SoC chemotherapy (*n* = 16), while those in the “hot” tumor group were randomized 1:1 to either SoC chemotherapy (*n* = 22) or durvalumab + tremelimumab (*n* = 23) before RC. Among the patients with TIS “hot” tumors, the rate of pCR in those receiving combined ICI (35%) was comparable to the NAC group (36%); patients with TIS “cold” tumors had a pCR rate of 69% (those treated with NAC). In this study, it was concluded that there was no clear advantage to selecting tumors for neoadjuvant immunotherapy based on the IFN-gamma immune signature. Finally, as previously mentioned, the phase 1b trial PrE0807 evaluated the feasibility of two doses of neoadjuvant nivolumab +/− lirilumab [68]. Of those who received ICI combination treatment (*n* = 30), 29 patients (97%) were able to proceed with RC; however, the pCR rate was only 18% and the proportion of patients who experienced pathologic downstaging was 29%.

In summary, the use of neoadjuvant ICI in patients with MIBC represents a promising treatment approach for those who are cis-ineligible, based on reported pCR rates. However, the benefit of an ICI doublet compared to SoC NAC remains unclear, in part due to the high rates of toxicities observed in major clinical trials. Furthermore, a notable finding in the MDACC trial was the significant association between preexisting immune infiltration and improved antitumor immune response (which was also seen in the PURE-01 and ABACUS trials), yet NABUCCO failed to demonstrate this. Potential reasons for these conflicting findings may be due to the inclusion of patients with more advanced disease in the NABUCCO and the trial’s smaller sample size [81].

Further investigation in larger phase 3 studies is needed to prove a survival benefit and identify novel biomarkers to improve patient selection before the widespread adoption of neoadjuvant immunotherapy in MIBC. For example, a recent study investigated RNA-sequencing-based immune subtypes in patients with MIBC treated with neoadjuvant ICIs, which may form the basis for future predictive biomarkers [82].

## 5. Neoadjuvant Immunotherapy in PC

### 5.1. Brief Overview of PC and Current Treatment Approaches

PC ranks as the second most prevalent cancer and the fifth leading cause of cancer-related mortality among males worldwide, posing a significant burden on global health [1]. Most newly diagnosed cases of PC are localized, in which the prognosis is favorable, boasting a 5-year survival rate exceeding 99% [83]. Treatment options for localized disease may include radical prostatectomy (RP), radiation therapy, or active surveillance, depending on a patient’s predetermined disease risk [5]. However, in men with high-risk localized PC—defined by a Gleason score of 8–10, prostate-specific antigen (PSA) levels greater than 20 ng/mL, or cT2c or higher—there is a heightened risk of disease recurrence after definitive treatment [6]. Up to 40% of these high-risk individuals may experience recurrence within 5 years [84]. This recurrence correlates with lower biochemical recurrence-free survival rates, estimated at 60–70% over 5 years. Furthermore, these patients typically exhibit reduced cancer-specific survival, with 10-year PC-specific survival rates ranging from 72–92% following RP [85].

To date, neoadjuvant systemic therapies such as androgen deprivation therapy (ADT) and chemotherapy have failed to demonstrate a significant clinical benefit in PC [86]. For instance, the CALGB 90203 trial evaluated the use of chemohormonal therapy (ADT + docetaxel) in patients with high-risk localized PC; however, it failed to demonstrate improved 3-year biochemical PFS over RP alone [87]. Several trials have also evaluated the use of neoadjuvant androgen receptor signaling inhibitor (ARSI) treatment combinations [88]. For instance, the phase 2 ARNEO trial evaluated the use of neoadjuvant degarelix +/− apalutamide in patients with high-risk PC [89]. While neoadjuvant ARSI combination treatment was associated with a significantly improved pathological response, there was no statistically significant difference in biochemical recurrence between the two groups at 3 years of follow-up. Currently, the phase 3 PROTEUS trial (NCT03767244) is evaluating the use of apalutamide in combination with ADT in patients with high-risk PC [90]. Although neoadjuvant trials thus far have failed to demonstrate a significant survival benefit, there has been growing interest in investigating neoadjuvant novel immunotherapies to improve outcomes in patients with high-risk localized PC.

### 5.2. Tumor Immunology and Immune Evasion Mechanisms in PC

Since the introduction of the first immunotherapy drug, sipuleucel-T, for metastatic castrate-resistant PC (mCRPC) in 2010, there has been a significant increase in immunotherapy trials for PC [91]. However, the efficacy of immunotherapy in PC has been limited thus far [92]. Unlike the significant improvements in survival seen with ICIs in RCC and bladder cancer, their effectiveness in PC has been more subdued [91]. The underlying reasons for this are likely multifactorial [92]. PC is considered less immunogenic than cancers like melanoma and non-small cell lung cancer (NSCLC) due to a lower TMB, resulting in fewer neoantigens that limit immune system recognition and response [93]. PC can sometimes provoke an immune response through specific antigens, but this is often insufficient for a robust antitumor reaction without intervention [93]. The tumor microenvironment (TME) further complicates immune engagement, characterized by immunosuppressive elements such as regulatory T cells (Tregs), myeloid-derived suppressor cells (MDSCs), and cytokines like transforming growth factor-beta (TGF-β) and interleukin-10 (IL-10), which inhibit the function of key immune cells including cytotoxic T lymphocytes and natural killer cells [94,95,96]. Furthermore, antigen presentation is often impaired in PC, with reduced major histocompatibility complex class I expression, thus impairing the efficacy of ICIs [97]. Collectively, these factors create a challenging environment for immunotherapy in PC [92], highlighting the need for innovative strategies to enhance treatment response. There is evidence to suggest that ADT has several immunostimulatory effects through increased T-cell infiltration, enhanced antigen presentation, thymic regeneration, and modulation of myeloid cells through expansion of pro-inflammatory tumor-associated macrophages [98]. Additionally, the emergence of AMG 509, known as xaluritamig, presents a promising avenue. Xaluritamig, a STEAP1-targeted T-cell engager, offers a novel mechanism of action that directs T-cell-mediated lysis of PC cells. Encouraging results were seen in a first-in-human study, particularly in late-line mCRPC patients. The study showed a 59% PSA50 response rate and a 41% ORR in high-dose cohorts [99].

### 5.3. Rationale for Investigating Neoadjuvant Immunotherapy in PC

Even though the effectiveness of immunotherapy in PC remains limited, recent advances in our understanding of the TME and immune evasion in PC have fueled interest in exploring neoadjuvant immunotherapy as an innovative treatment approach. Neoadjuvant immunotherapy seeks to modify the immunosuppressive TME characteristic of cancer, enhancing immune system activation for better tumor cell recognition and destruction before and after surgery [100]. This strategy offers the potential for tumor downstaging before primary treatments like surgery or RT, possibly resulting in less invasive procedures and improvement in patient outcomes [100]. It also aims to boost tumor immunogenicity by increasing the release of tumor antigens and improving antigen presentation, thereby fostering stronger and more durable immune responses after surgery [101]. As the PC treatment landscape continues to evolve, particularly for advanced-stage disease, the investigation of neoadjuvant immunotherapy represents a significant and promising research avenue, essential for creating approaches that effectively harness the immune system to combat this disease.

### 5.4. Clinical Trials of Neoadjuvant Immunotherapy in PC (Table 3)

Sipuleucel-T is an active cellular immunotherapy, which enhances the patient’s immune response against PC by activating dendritic cells with a fusion protein targeting prostatic acid phosphatase [102]. It was approved by the US Food and Drug Administration (FDA) based on the results of the phase 3 IMPACT trial, which demonstrated a significant reduction in mortality risk and OS benefit in mCRPC patients [103]. A phase 2 study evaluated tissue samples (obtained pre- and post-treatment) of patients with localized PC who were treated with three doses of sipuleucel-T given at the standard 2-week intervals before RP. The purpose of this study was to examine the effects of treatment on the TME [104]. The findings revealed that neoadjuvant sipuleucel-T treatment markedly increased T-cell presence in the prostate, particularly where benign and malignant glands meet, thus altering lymphocyte distribution within PC tissue [104]. This approach not only stimulated T helper 1 (Th1) immune responses and regulated the tumor’s immune environment, but also enhanced Th1-associated gene expression and T-cell infiltration, notably CD8+ T cells driven by C-X-C motif chemokine ligand 10, without markedly affecting the Th2, Th17, or Treg genes [104]. Decreases in serum PSA levels were linked to Th1 response induction, suggesting an immunological benefit, whereas PSA level increases were tied to the upregulation of inhibitory checkpoint genes like CTLA-4, carcinoembryonic antigen-related cell adhesion molecule 6, and the T-cell immunoglobulin and immunoreceptor tyrosine-based inhibitory motif (ITIM) domain, indicating potential therapy-induced adaptive immune resistance mechanisms [104]. These insights underscore the complex interplay of immune activation and suppression in sipuleucel-T treatment for PC, highlighting both the potential and the challenges of improving immunotherapy outcomes by overcoming immune resistance [104]. Next, a phase 2 study evaluated the use of neoadjuvant docetaxel + GVAX, a granulocyte-macrophage-CSF (GM-CSF)-secreting allogeneic cellular vaccine, in six patients with high-risk localized PC before RP [105]. Patients received docetaxel 75 mg/m^2^ every 3 weeks for four cycles, and GVAX was given 2–3 days following each chemotherapy cycle. After RP, patients were treated with an additional six doses of GVAX. Five patients (83%) successfully underwent RP, and four had downstaging of their Gleason score. Additionally, a phase 2 study evaluated the use of neoadjuvant cyclophosphamide (Cy)/GVAX + ADT prior to RP in 29 patients with high-risk localized PC (cT1c-T3bN0, and a Gleason score ≥ 4 + 3) [106]. Patients were randomized 1:1 to ADT alone and intravenous Cy 200 mg/m^2^ and GVAX given 2 weeks before ADT with additional comparison to a control group who underwent immediate RP. In this study, treatment with both Cy/GVAX + ADT and ADT alone led to significant increases in intratumoral CD8+ T-cell infiltration and PD-L1 expression compared to the control group, indicating the immunostimulatory potential of androgen ablation. However, the CD8+ T-cell infiltrate was accompanied by a proportional increase in Tregs, which may dampen the immunogenicity of ADT [106]. In general, data from clinical trials in this area remain limited. For example, a phase 3 trial (NCT00577356) examining docetaxel + CG1940/CG8711 (immunotherapy drugs) given before RP in high-risk PC was terminated due to safety concerns [107]. Additionally, a phase 2 study that sought to investigate the use of leuvectin, a plasmid DNA expression vector encoding IL-2, followed by surgery in patients with stage II/III PC (NCT00004050) was terminated [108]. Finally, a phase 2 study (NCT04020094), which explored the potential synergistic effect of atezolizumab + PROSTVAC (a vector-based therapeutic vaccine against PSA-expressing tumor cells), was also suspended prematurely [109].

In summary, data from these trials underscore the modest immune modulation and antitumor activity achieved with traditional vaccines and ICIs in PC. These results suggest that more robust modalities such as T-cell engagers or innovative combination therapies may be required to enhance the efficacy of immunotherapy in this setting.

**Table 3:** Summary of prospective neoadjuvant immunotherapy trials in high-risk PC.

NCT Number	Pt Selection	Phase	Est. pts	Trial Title	Sponsor	Ref.
NCT00577356	Clinical stage I-III, M0	II	6	Docetaxel + Immunotherapy Prior to RP for High-Risk PC	Benaroya Research Institute	[107]
NCT00089856	Localized PC planned for RP	II	6	Neoadjuvant docetaxel + GVAX^®^ followed by RP in pts with high-risk clinically localized PC	Cell Genesys	[105]
NCT00004050	Clinical stage II/III, Gleason score ≥ 6	II	13	Leuvectin Followed By Surgery in Tx of pts With Stage II/III PC	Vical	[108]
NCT04020094	Clinical stage unfavorable int-, high-risk or very high-risk PC	II	0	Periop Atezo + MVA-BN-Brachyury + PROSTVAC^®^ For Int- And High-Risk Localized PC (AtezoVax)	University of Utah	[109]
NCT00715104	Localized PC planned for RP	II	42	Neoadjuvant Sipuleucel-T in pts With Localized PC	UCSF	[104]
NCT01696877	cT1c-T3bN0M0	Ib/II	29	Neoadjuvant Androgen Ablation + Cy + GVAX^®^ Vaccine for Localized PC	Hopkins	[106]

Abbreviations: NCT, national clinical trial; pt, patient; est, estimated; ref, reference; RP, radical prostatectomy; PC, prostate cancer; GVAX, GM-CSF secreting vaccine; Tx, treatment; int, intermediate; Periop, perioperative; atezo, atezolizumab; PROSTVAC, vector-based therapeutic vaccine against PSA-expressing tumor cells; Cy, cyclophosphamide.

## 6. Neoadjuvant Immunotherapy in Penile/Testicular Cancers

### 6.1. Penile Squamous Cell Carcinoma

PSCC is a rare GU malignancy, with an incidence of <1 case per 100,000 men [110]. In patients with more advanced disease (pN3 or M1), survival outcomes remain poor with limited effective treatment options [111]. High-risk PSCC is defined by the presence of certain histologic subtypes (sarcomatoid, basaloid, and adenosquamous carcinomas) and lymph node involvement [112]. Human papillomavirus positivity is associated with a better prognosis [113]. Nevertheless, the 5-year OS rate among patients with inguinal or lymph node metastasis is dismal, ranging from 10–20% [111]. Neoadjuvant chemotherapy with TIP (Paclitaxel + Ifosfamide + Cisplatin) is the SoC before inguinal lymph node dissection in patients with ≥4-cm inguinal lymph nodes [112]. Preclinical work has demonstrated increased rates of tumor-infiltrating immune cells and high PD-L1 expression (40–60%) in PSCC, which suggests that further evaluation of ICI is warranted [111].

Currently, there are limited prospective data evaluating the use of ICIs in PSCC. For example, in a phase 2 study of patients with rare, advanced GU cancers, five patients with PSCC failed to respond to combination nivolumab and ipilimumab therapy [114]. Additionally, in a case series of a phase 2 basket trial evaluating the use of pembrolizumab in patients with advanced PSCC, two patients experienced PD, and one patient with microsatellite instability-high (MSI-H) disease exhibited a PR [115]. Finally, a phase 2 trial (NCT03686332) evaluated 32 patients with advanced PSCC treated with atezolizumab every 3 weeks (for up to 1 year) +/− RT [116]. Antitumor activity was observed in advanced PSCC; however, the primary endpoint was not met, as the 1-year PFS was 12% (95% CI, 4.0–33%), which was below the prespecified target of ≥35% [116]. In summary, the activity of ICI monotherapy in advanced PSCC remains limited. The best evidence for its use is from a multicenter retrospective study: among eighty-five evaluable patients with advanced PSCC, an ORR of 14% was observed, including two patients who achieved a CR and four patients who had a PR [117]. Further studies are needed to evaluate which patients would derive the most benefit from ICI. This has prompted investigation into the role of neoadjuvant ICI combination therapies for the treatment of high-risk PSCC.

### 6.2. Testicular Germ Cell Tumors

TGCTs are often highly responsive to platinum-based chemotherapy, with an 80% cure rate in those with metastatic disease. Unfortunately, for those who progress following chemotherapy, outcomes remain poor [118]. ICIs have been evaluated in chemotherapy-refractory disease; however, meaningful response rates have not been observed in case series and early-phase clinical trials [119,120,121]. Reasons for a poor response rate include an immunosuppressive TME and a low TMB [122].

More recently, there have been several studies evaluating claudin-6 (CLDN-6), a tight junction protein, which is abnormally expressed in several cancers, as a potential therapeutic target. A phase 2 study evaluated the use of a CLDN-6 monoclonal antibody (mAb) in patients with relapsed/refractory TGCTs; however, the study was terminated early due to lack of efficacy [123]. Additionally, there is an ongoing phase 1/2 trial (NCT 05262530) evaluating the use of a bispecific antibody targeting CLDN-6 and CD3 [124]. Finally, an ongoing phase 1/2 trial (NCT04503278) is evaluating the use of CLDN-6 chimeric antigen receptor (CAR) T-cell therapy +/− an investigational RNA-based vaccine in patients with CLDN-6-positive solid tumors [125]. An interim analysis of 22 patients treated at two dose levels demonstrated an ORR of 33% in 21 evaluable patients, including one CR.

In summary, there remains a lack of data regarding the use of ICIs in the neoadjuvant setting for patients with TGCTs. To date, studies have been limited to those with metastatic relapsed/refractory disease, for which ICIs have not demonstrated significant antitumor activity.

## 7. Cost-Effectiveness of Neoadjuvant Immunotherapy in GU Cancers

While the efficacy of neoadjuvant immunotherapy in GU cancers remains an active area of research, the cost of these treatments must also be considered, especially in resource-limited areas. One study analyzed the cost-effectiveness of neoadjuvant immunotherapy in UC and found that neoadjuvant ICIs were not cost-effective (except for atezolizumab) compared to cisplatin-based chemotherapy [126]. Additionally, several studies have evaluated the cost-effectiveness of neoadjuvant ICIs for other cancer types, including breast and lung cancer. For example, neoadjuvant chemoimmunotherapy has been successfully incorporated into the SoC of early-stage triple-negative breast cancer (TNBC), followed by adjuvant immunotherapy, as it has been shown to improve pCR rates, EFS, and OS [127]. A cost analysis of neoadjuvant chemoimmunotherapy was performed in patients with early-stage TNBC and found significant improvements in quality-adjusted life years (QALYs) compared to chemotherapy alone [128]. Additionally, neoadjuvant chemoimmunotherapy has been shown to improve EFS, pCR, and major pathological response in patients with resectable NSCLC [129,130]. Utilizing data from the phase 3 KEYNOTE-671 trial, a cost analysis study found perioperative pembrolizumab to be a cost-effective option in patients with resectable NSCLC as there were improvements seen in QALYs compared to NAC [131].

## 8. Limitations of Neoadjuvant Chemotherapy in Patients with Urothelial Carcinoma

As previously stated, neoadjuvant cisplatin-based NAC is the SoC for certain patients with MIBC. However, the use of NAC among eligible patients remains underutilized, with a reported range of 15–40% [132]. One possible reason for the underutilization of NAC may be the perceived increased risk of short-term postoperative complications and post-hospital mortality. However, data from several retrospective studies do not support these findings [132,133,134,135,136]. Another potential reason is the perception that older patients may experience increased toxicity following treatment with NAC due to the presence of several comorbidities [137]; however, several studies have demonstrated that NAC is well tolerated in certain older patients and the outcomes are comparable to that of younger patients [132,133,134,135,136]. Finally, the possibility of missing the window of curative treatment may also be a reason for the underutilization of NAC; however, the data also do not support this, as it has previously been shown that a ddMVAC regimen given within a relatively short period of time can be equally as effective and less toxic compared to a standard GC treatment regimen [138].

## 9. Future Directions

### 9.1. Ongoing Neoadjuvant Immunotherapy Trials in GU Cancers (Table 4)

#### 9.1.1. Ongoing RCC Trials

The use of neoadjuvant immunotherapy in RCC remains an active area of investigation, with a focus on novel immunotherapy combination treatments. The SPARC-1 trial (NCT04028245) is a phase 2 trial investigating the use of two doses of neoadjuvant spartalizumab (anti-PD-1) and canakinumab (IL-1β mAb) in patients with localized ccRCC (cT1b-T4Nany or cTanyN1) [139]. The triple-arm NESCIO trial (NCT05148546) is evaluating the use of neoadjuvant nivolumab, nivolumab + ipilimumab, or nivolumab + ipilimumab + relatlimab (anti-lymphocyte-activation-gene-3) in localized ccRCC (cT1b-T2a grade 4, N0; cT2b grade 3, N0; cT3 any grade, N0; cT4 any grade, N0; cTanyN1). Patients will be randomized 1:1:1 and will be treated for 6 weeks before surgery [140]. Additionally, the NAPSTER trial (NCT05024318) is evaluating stereotactic RT +/− 3 cycles of pembrolizumab in patients with localized ccRCC (cT1b-T3 or N1+; or low volume M1) prior to nephrectomy [141]. Next, a phase 2 neoadjuvant trial is investigating lenvatinib + pembrolizumab (NCT05319015) for 12 weeks in patients with RCC and IVC tumor thrombus (cT3-4N0-1M0-1) [142]. Finally, another trial (NCT05733715) is investigating lenvatinib + pembrolizumab (one 3-week cycle; up to eight 6-week cycles) in patients with localized RCC (cT2-T4) [143].

**Table 4:** Ongoing selected immunotherapy trials in patients with GU malignancies.

NCT Number	Cancer Type	Pt Selection	Phase	Est. pts	Trial Title	Sponsor	Ref.
NCT05148546	RCC	cT1b-T2aN0; cT2bN0; cT3N0; cT4N0; cTanyN1	II	69	Neoadjuvant Combo IO for Primary ccRCC	The Netherlands Cancer Institute	[140]
NCT05319015	RCC	cT3-4,N0-1,M0-1	II	30	Neoadjuvant Lenvatinib + Pembro for IVC TT	UTSW	[142]
NCT05733715	RCC	cT2-T4	I	30	Neoadjuvant Pembro + Lenvatinib for RCC	UPenn	[143]
NCT05024318	RCC	cT1b-T3N0; or N1M0; or low volume M1	II	20	NeoAdjuvant Pembro + STEreotactic RT Prior to Nephrectomy for RCC	Peter MacCallum Cancer Centre	[141]
NCT06138496	RCC	cT1b with an endophytic component ≥ 75%; or cT2	II	43	Cadonilimab + Lenvatinib as Neoadjuvant Tx for ccRCC	Sun Yat-sen University	[144]
NCT03341845	RCC	cT1b-4N0-1	II	40	Neoadjuvant axitinib + avelumab for Pts With Localized ccRCC	The Netherlands Cancer Institute	[43]
NCT04028245	RCC	cT1b-T4Nany	I	14	A Study of Spartalizumab + Canakinumab in Pts With Localized ccRCC	Columbia University	[139]
NCT06511648	UC	cT2-T4aN0/N1	II	90	Erdafitinib +/− Cetrelimab in MIBC Pts With FGFR Gene Alterations	Spanish Oncology GU Group	[145]
NCT03924856	UC	cT2-T4aN0; or cT1-T4aN1	III	907	Periop Pembro + Neoadjuvant Chemo vs. Periop Placebo + Neoadjuvant Chemo for Cis-eligible MIBC	Merck Sharp & Dohme	[146]
NCT04700124	UC	cT2-T4aN0; or cT1-T4aN1	III	784	Periop EV + Pembro vs. Neoadjuvant Chemo for Cis-eligible MIBC	Merck Sharp & Dohme	[147]
NCT03924895	UC	cT2-T4aN0; or cT1-T4aN1	III	857	Periop Pembro + RC or Periop Pembro + EV + RC vs. RC Alone in Pts Who Are Cis-ineligible or Decline Cisplatin With MIBC	Merck Sharp & Dohme	[148]
NCT02933255	PC	Localized PC planned for RP	I/II	24	PROSTVAC^®^ + Nivo in Men With PC	NCI	[149]
NCT06555796	PC	Biochemically recurrent PC after definitive Tx by either RP or XRT	Ib	40	Evaluation of Xaluritamig in High-Risk, Biochemically Recurrent, nmCSPC	Amgen	[150]
NCT04301414	PC	cT1c-T3b, N0	I	26	Non-fucosylated Anti-CTLA-4 + Degarelix vs. Degarelix Alone in Men With High-risk Localized PC	Columbia University	[151]
NCT03821246	PC	Int-high risk PC	II	68	Neoadjuvant Atezo-Based Combination Therapy in Men With Localized PC Prior to RP	UCSF	[152]
NCT04009967	PC	Non-metastatic PC with Gleason grade ≥ 8	II	30	Biomarkers for Neoadjuvant Pembro in Non-mPC Positive by 18FDG-PET Scanning	CHU de Quebec-Universite Laval	[153]
NCT03899987	PC	Localized PC planned for RP	II	30	Aspirin + Rintatolimod +/− IFN-alpha 2b in Tx Pts With PC Before Surgery	Roswell Park	[154]
NCT04475016	PSCC	cT4anyNany; or cTanyN3	II	29	TIP + Nimotuzumab and Triprilimab as Neoadjuvant Tx in LA Penile Cancer	Sun Yat-sen University	[155]
NCT05262530	TGCT	CLDN6-positive solid tumors	I/II	330	Safety and Prelim Efficacy Trial of BNT142 in Pts With CLDN6-positive Solid Tumors	BioNTech SE	[124]

Abbreviations: NCT, national clinical trial; pt, patient; est, estimated; ref, reference; IO, immuno-oncology; ccRCC, clear-cell renal cell carcinoma; pembro, pembrolizumab; IVC, inferior vena cava; TT, tumor thrombus; MIBC, muscle-invasive bladder cancer; FGFR, fibroblast growth factor receptor; Vs, versus; chemo, chemotherapy; EV, enfortumab vedotin; PROSTVAC, vector-based therapeutic vaccine against PSA-expressing tumor cells; nivo, nivolumab; PC, prostate cancer; CTLA-4, cytotoxic lymphocyte antigen-4; int, intermediate; atezo, atezolizumab; RP, radical prostatectomy; XRT, external beam radiation; nmCSPC, non-metastatic castrate-sensitive PC; mPC, metastatic PC; 18FDG-PET, 2-deoxy-2-[fluorine-18] fluoro-D-glucose positron emission tomography; IFN, interferon; TIP, Paclitaxel + Ifosfamide + Cisplatin; PSCC, penile squamous cell carcinoma; LA, locally advanced; CLDN6, claudin-6; TGCTs, testicular germ cell tumors.

#### 9.1.2. Ongoing UC Trials

There are several ongoing perioperative (neoadjuvant and adjuvant) trials in UC. Erdafitinib is a fibroblast growth factor receptor 3 (FGFR3) inhibitor, which is currently approved for mUC [156]. A phase 2 trial (NCT06511648) is investigating the neoadjuvant use of erdafinitib +/− cetrelimab (anti-PD-1) for cis-ineligible patients with MIBC and FGFR gene alterations (cT2-4aN0/N1) [145]. Next, a phase 2 trial (NCT06263153) is evaluating neoadjuvant futibatinib, an inhibitor of FGFR1–4, in combination with durvalumab for cis-ineligible patients with MIBC and FGFR overexpression (cT2-T4aN0) [157]. Cis-eligible patients with MIBC are being studied in these phase 3 trials: ENERGIZE (NCT03661320; GC + nivolumab +/− linrodostat mesylate [indoleamine 2,3-dioxygenase 1 inhibitor]) and KEYNOTE-866 (NCT03924856; GC + pembrolizumab) [146,158]. Finally, the recent FDA approval of several antibody-drug conjugates (ADCs) in mUC has accelerated their inclusion in the perioperative space. The novel ADC enfortumab vedotin (EV), which targets nectin-4 cells and is paired with a microtubule inhibitor conjugate, in combination with pembrolizumab has shown unprecedented response rates and improvement in OS in the metastatic and locally advanced settings [159]. Phase 3 trials of EV + pembrolizumab in the neoadjuvant setting (NCT04700124, NCT03924895, NCT04960709) are currently accruing patients and will define the potential role of this regimen in the perioperative space [147,148,160]. The VOLGA trial is evaluating the use of durvalumab + tremelimumab + EV compared to durvalumab + EV as a neoadjuvant therapy for cis-ineligible patients with MIBC (cT2-4aN0-N1) [161]. In summary, there is excitement within the field with ADC combination therapy in MIBC as it offers a possibility of a durable response with a tolerable risk profile.

#### 9.1.3. Ongoing PC Trials

Despite the challenges with neoadjuvant immunotherapy for PC, the search for effective treatments continues. There is preliminary evidence suggesting that immunotherapy may synergize with traditional treatments like ADT and RT [162,163], potentially amplifying antitumor effects and leading to better outcomes. Therefore, several ongoing trials are investigating innovative strategies that integrate immunotherapy with other treatments such as vaccines, chemotherapy, or ADT in the neoadjuvant setting. These combination approaches aim to counteract the immunosuppressive nature of the TME, boost tumor immunogenicity, and elicit stronger, more durable immune responses perioperatively. For example, a phase 1 trial (NCT04301414) is investigating the use of neoadjuvant degarelix acetate +/− non-fucosylated anti-CTLA-4 (BMS-986218) in men with high-risk localized PC (cT1c-T3bN0, Gleason sum of ≥4 + 3) [151]. Additionally, a phase 2 trial (NCT03821246) is examining an atezolizumab-based combination with tocilizumab or etrumadenant for men with localized PC before RP [152]. Finally, the phase 1/2 NEPI trial will investigate neoadjuvant treatment with the radionuclide lutetium-177 vipivotide tetraxetan prostate-specific membrane antigen +/− ipilimumab in patients with very-high-risk PC (International Society of Urological Pathology-Gleason Grade 4 + 5 and cT3 plus cN+ or PSA > 20 ng/mL) who are candidates for RP [164].

#### 9.1.4. Ongoing PSCC Trials

Currently, a phase 2 trial (NCT04475016) is investigating the use of neoadjuvant TIP + nimotuzumab (epidermal growth factor receptor [EGFR] mAb) + triprilimab (anti-PD-1) in patients with locally advanced PSCC (cT4Nany or cTanyN3) [155]. Another phase 2 single-arm trial (NCT04224740) is evaluating the use of neoadjuvant pembrolizumab + cisplatin-based chemotherapy as a first-line systemic therapy in patients with advanced PSCC [165].

### 9.2. Identifying Strategies to Improve Patient Selection in Neoadjuvant Immunotherapy (Table 5)

#### 9.2.1. RCC Predictive Biomarkers

Neoadjuvant immunotherapy clinical trials provide an opportunity for the discovery of biomarkers and a personalized treatment approach in GU malignancies [33]. Unlike other cancers, PD-L1 expression and TMB have failed to predict ICI treatment response in RCC [166]. Hence, there is an increased interest in the development of validated RCC biomarkers that could potentially guide ICI treatment selection and prognostication [167]. One potential RCC biomarker is the presence of sarcomatoid features upon histopathological examination [168]. Sarcomatoid features have been associated with an improved response to ICI in several trials in patients with advanced RCC including Checkmate-214 and IMmotion-151 [169,170,171]. Another potential RCC biomarker is the degree of CD8+ T-cell infiltration within tumors; higher levels have been associated with an improved ICI response in other cancers such as melanoma [168]. In the JAVELIN Renal 101 trial, patients with higher levels of CD8+ T cells at the invasive tumor margin experienced improved PFS following treatment with axitinib + avelumab [172]. Molecular subtyping and transcriptional analysis of tumors represent other potential emerging RCC biomarker options. For instance, in the IMmotion 150 trial, which investigated the use of atezolizumab +/− bevacizumab (anti-vascular endothelial growth factor) in untreated mRCC patients, three inflammatory gene signatures were created. Interestingly, the T-effector-cell gene signature was associated with increased PD-L1 expression and CD8+ T-cell infiltration, as well as an improved ICI response [173]. Additionally, loss-of-function mutations in the Polybromo-1 (PRBM1) gene have been associated with an improved ICI response in RCC in several studies [168]. Finally, a study presented at the 2024 ASCO GU conference investigated the role of kidney injury molecule-1 (KIM-1) as a potential biomarker for patients with localized RCC at high risk for recurrence post-nephrectomy who may benefit from adjuvant atezolizumab treatment [174]. High baseline serum KIM-1 levels were associated with a worse prognosis but improved response to atezolizumab compared to the placebo.

**Table 5:** Potential neoadjuvant immunotherapy biomarkers for patients with GU malignancies.

Biomarker	Cancer Type(s)	Testing Method	ICI Response	Ref.
PD-L1 expression	UC	IHC	Role is unclear	[168]
TMB	UC	NGS	Higher TMB trends toward improved responses; however, not statistically significant	[168,175]
Sarcomatoid features	RCC	Histopathologic examination	Improved	[169,170,171]
CD8 T cell infiltration	RCC, UC	IHC	Improved	[62,64,71,172]
ctDNA clearance	UC	NGS	Improved	[70]
Tertiary lymphoid structure	UC	IHC	Improved	[70]
Molecular subtyping/Gene expression panels	RCC, UC	NGS	May have increased predictive ability for ICI response	[82,173,176]
PRBM1 mutation	RCC	NGS	Improved	[168]

Abbreviations: ICI, immune checkpoint inhibitor; PD-L1, programmed death-ligand 1; UC, urothelial carcinoma; IHC, immunohistochemistry; TMB, tumor mutational burden; NGS, next generation sequencing; RCC, renal cell carcinoma; CD8, cluster of differentiation 8; ctDNA, circulating tumor DNA; PRBM1, Polybromo-1.

#### 9.2.2. UC Predictive Biomarkers

Like RCC, several studies have evaluated the use of PD-L1 expression as a potential predictive biomarker in UC. High PD-L1 expression enriches for favorable responses to ICIs in UC, but it is not a good discriminating biomarker in UC and thus is not used clinically [168]. Next, TMB has been extensively studied as a biomarker in UC, which may potentially enhance ICI treatment responses [168]. For instance, in an exploratory analysis of the IMVigor210 Cohort II, ICI responders had an increased TMB compared to non-responders [168]. Furthermore, in IMVigor 210 Cohort 1, patients in the highest quartile of TMB had a significantly prolonged mOS [175]. While these results regarding TMB are encouraging, further strategies are needed to improve standardization in measuring and reporting TMB, which may improve its utility as a reliable biomarker for ICI response in UC. Additionally, the degree of tumor immune infiltration has also been identified as having potential prognostic and predictive value in patients with UC [177]. For example, in the ABACUS, PURE-01, and MDACC trials, higher levels of CD8+ T-cell infiltration within tumors were associated with a significant response to a neoadjuvant immune checkpoint blockade [62,64,71]. However, in the NABUCCO trial, baseline CD8+ immune cell infiltration was not associated with a treatment response, but tertiary lymphoid structures and circulating tumor DNA (ctDNA) clearance were [70]. One potential reason for the discordant results may be due to the lack of standardization in measuring immune infiltration [81]. Molecular subtyping of MIBC (based upon The Cancer Genome Atlas (TCGA) and genomic subtyping classifier models) and immune signatures have also been evaluated as promising biomarkers of response to neoadjuvant immunotherapy. In the PURE-01 trial, patients with the claudin-low MIBC molecular subtype appeared to have the best outcomes after neoadjuvant immunotherapy [176]. Furthermore, in a separate analysis of the PURE-01 trial, five transcriptomic subtypes were found to be associated with clinical and pathological responses to pembrolizumab [82]. Specifically, tumors with enhanced IFN-A and IFN signatures demonstrated a better response than tumors with low IFN expression. Finally, bulk and single-cell RNA sequencing were used to investigate the potentially predictive role of the Ascore, an anoikis-based four-gene prognostic signature for predicting PD and immunotherapy response in bladder cancer. Anoikis is a specific form of apoptosis where cells detach from the extracellular matrix or surrounding cells. The Ascore demonstrated superior predictive capacity for bladder cancer ICI responses compared to traditional biomarkers like TMB and PD-L1 [178]. However, further studies will be needed to validate these findings.

#### 9.2.3. PC Predictive Biomarkers

As previously mentioned, the effectiveness of ICIs in PC thus far has been limited, likely due to several factors, including a low TMB and an immunosuppressive TME. Studies such as KEYNOTE-028 have suggested an improved response to pembrolizumab in a subset of patients with mCRPC (MSI-H disease, mismatch repair deficiency, high TMB, and DNA polymerase epsilon mutations) [168]. Additionally, a retrospective study demonstrated the clinical activity of anti-PD-1 ICIs in the treatment of patients with cyclin-dependent kinase 12 mutated advanced PC [179]. Further investigation is needed to identify reliable biomarkers for ICI responses in PC. For example, a phase 2 study (NCT04009967) is currently recruiting participants to investigate biomarkers for neoadjuvant pembrolizumab in non-metastatic PC [153].

## 10. Conclusions

In summary, the utilization of neoadjuvant immunotherapy in GU malignancies remains an active area of investigation. While results from neoadjuvant studies in RCC and UC appear promising, we need to demonstrate an OS benefit in phase 3 trials compared to the current SoC. Additionally, given the limited success of ICIs in PC thus far, the discovery and validation of effective biomarkers for ICI responders and novel immunotherapy agents such as bispecific T-cell engagers will undoubtedly be crucial in optimizing patient selection and improving treatment outcomes. Several unanswered questions remain, which future neoadjuvant immunotherapy studies in GU cancers will hopefully address, such as determining the optimal timing and sequence of immunotherapy, identifying the most effective immunotherapeutic agents, improving our understanding of the TME, and designing clinical trials that accurately assess the effectiveness of these therapies. More specifically, neoadjuvant ICI clinical trials offer a unique opportunity for longitudinal analysis of biological specimens collected from patients (blood, urine, tissue obtained via biopsy), utilizing various technologies such as DNA/RNA sequencing and ctDNA and cell-free DNA testing, which can then be paired with analysis of tissue obtained following surgery to determine ICI treatment responses and mechanisms of resistance.
